# Prevalence of Oropharyngeal Candidiasis and distribution of *Candida* species among People Living with Human Immunodeficiency Virus in Africa: a systematic review and meta-analysis

**DOI:** 10.21203/rs.3.rs-4534730/v1

**Published:** 2024-06-06

**Authors:** Benson Musinguzi, Ekwaro A. Obuku, Alison Annet Kinengyere, Regina Ndagire, Andrew Baguma, Alex Mwesigwa, Herbert Itabangi, Gerald Mboowa, Obondo James Sande, Beatrice Achan

**Affiliations:** Department of Medical Laboratory Science, Faculty of Health Sciences, Muni University, Arua, Uganda; Africa Centre for Systematic Reviews and Knowledge Translation, College of Health Sciences, Makerere University, Kampala, Uganda; Sir Albert Cook Medical Library, College of Health Sciences, Makerere University, Kampala Uganda; Clinical Epidemiology Unit, School of Medicine, College of Health Sciences, Makerere University, Kampala, Uganda; Department of Microbiology, School of Medicine, Kabale University, Kabale, Uganda; Department of Microbiology, School of Medicine, Kabale University, Kabale, Uganda; Department of Microbiology and Immunology, Faculty of Health Sciences, Busitema University, Mbale, Uganda; African Centre of Excellence in Bioinformatics and Data Intensive, Sciences, the Infectious Diseases Institute, College of Health Sciences, Makerere University, Kampala, Uganda; Department of Immunology and Molecular Biology, School of Biomedical Sciences, College of Health Sciences, Makerere University, Kampala, Uganda; Department of Medical Microbiology, School of Biomedical Sciences, College of Health Sciences, Makerere University, Kampala, Uganda

**Keywords:** Candida, Candida albicans, Candida spp., HIV

## Abstract

**Background:**

The incidence of oropharyngeal candidiasis among people living with human immunodeficiency virus in Africa is on the rise. Oropharyngeal candidiasis is mainly caused by *C.albicans*; however, a shift in the etiology towards non-*Candida albicans* species is increasing. In addition, there are variations in the epidemiological distribution of *Candida* species causing oropharyngeal candidiasis among people living with human immunodeficiency virus in Africa.

**Objective:**

This review aimed to determine the prevalence of oropharyngeal candidiasis and the distribution of *Candida* species among people living with human immunodeficiency virus in Africa.

**Materials and Methods:**

This systematic review protocol was registered in the base PROSPERO database prior to its conduct (CRD42021254473). The Preferred Reporting Items for Systematic Reviews and Meta-Analyses Protocol guidelines (PRISMA-P) were followed for this study. The PubMed, Scopus and EMBASE databases were searched to identify published studies published between 1^st^ January 2000 and 8^th^ October 2022. The eligible studies were included in the meta-analysis and analyzed using a random effects model. The risk of bias of the included studies was assessed using the Joanna Briggs Institute quality assessment tool for prevalence studies.

**Results:**

The database search yielded 370 titles from PubMed (n=192), EMBASE (n=162) and SCOPUS (n=16). Fourteen studies with a total of 3,863 participants were included in the meta-analysis. The pooled prevalence of oropharyngeal candidiasis was 49.0% (95% CI: 37% - 62%). A total of 2,688 *Candida* isolates were reported; approximately 76.6% (n=2,060) were *C. albicans*, and 21.7% (n=582) were non-*C. albicans*. Among the non-*Candida albicans* species, *C*. *glabrata* was the most common isolate (29.6%), followed by *C*. *tropicalis* (27.7%), *C*. *krusei* (17.0%), *C*. *parapsilosis* (8.1%) and *C*. *dubliniensis* (5.2%). Out of 14 studies, 7 (50.0%) had a low risk of bias, 5 (35.7%) had a moderate risk of bias, and 2 (14.3%) had a high risk of bias.

**Conclusion:**

Almost half of people living with HIV in Africa have oropharyngeal candidiasis, and *C. albicans* remains the most frequent cause of oropharyngeal candidiasis.

## INTRODUCTION

Approximately 38 million people are living with human immunodeficiency virus (PLHIV) globally, 68% (25.7 million) of which are PLHIV (two-thirds) from Africa (1). The burden of oropharyngeal candidiasis (OPC) continues to increase, and it is the most prevalent opportunistic fungal disease associated with human immunodeficiency virus/acquired immunodeficiency syndrome (HIV/AIDS) (2–4).

The incidence of OPC and its etiological *Candida* species among PLHIV in Africa is increasing (2). The most common *Candida* species causing OPC among PLHIV include *Candida albicans* and Non *albicans candida* (NAC), e.g., *C. parapsilosis*, *C. glabrata*, *C. tropicalis*, *C. dubliniensis*, *C. krusei* and *C. guilliermondii* (5). *Candida albicans* is a common cause of OPC; however, over the years, non-albicans *Candida* species (NACs) have been isolated with increasing frequency among individuals with HIV/AIDS (6). Indeed, in one study of 315 HIV/AIDS patients with OPC in southwestern Uganda, *Candida albicans* accounted for 87%, *C. glabrata* 3.6%, *C. tropicalis* 3.6% and *C. norvegensis* 2.9% with OPC (7). Furthermore, multiple fungal-resistant Candida auris strains have been reported as rapidly emerging causes of nosocomial multidrug-resistant fungal diseases worldwide (8, 9).

The diagnosis of OPC is often based on clinical presentation, however, its empirical management is no longer adequate due to the varied antifungal susceptibility profiles of the different *Candida* species causing the mycosis. This consideration has gained clinical significance because of a shift in the etiology towards antifungal-resistant NAC species (10–12). For instance, the emergence of difficult-to-differentiate multidrug-resistant NAC species, including *C. dubliniensis*, *C. krusei*, *C. haemulonii* and *C. famata*, is increasing, as data on their epidemiological distributions in different African countries are still needed (8, 9, 13).

Limited data on the distribution of *Candida* species causing OPC coupled with the emergence of NAC have highlighted the importance of determining the distribution of *Candida* species and the prevalence of OPC among people living with HIV in Africa(14). This information is vital not only for proper OPC diagnosis and management among this vulnerable cohort of people in Africa but also for epidemiological purposes. Thus, this study reviewed the literature describing the prevalence of oropharyngeal candidiasis and the distribution of *Candida* species among people living with HIV in Africa.

## MATERIALS AND METHODS

### Study design

This systematic review and meta-analysis was conducted according to the PRISMA guidelines (15). The protocol of this review was registered in the open access PROSPERO database prior to conducting the review, number CRD42021254473 (https://www.crd.york.ac.uk/prospero/).

### Data sources

With the help of experienced libraries and information scientists, the PubMed (https://pubmed.ncbi.nlm.nih.gov/), Scopus (https://www.scopus.com/home.uri), and EMBASE (https://www.embase.com) databases were searched for relevant English-language articles. In addition, the reference lists of all identified studies were searched for relevant articles, and gray literature was searched for in Google Scholar (https://scholar.google.com/). The search was restricted to the period from 1^st^ January 2000 to 8^th^ October 2022. All the articles were exported to Mendeley Desktop 1.19.8 software (Mendeley Ltd., London, UK) for further processing, and duplicates were removed.

### Search strategy

The search terms were combined using Boolean operators OR for synonyms and ‘AND’ across elements of PICOS (population, intervention, comparison, outcome and study design), as follows:

The terms for the population of interest were ‘HIV’, ‘AIDS’, ‘human immunodeficiency virus’, and ‘acquired immune deficiency syndrome’. This population was restricted to sub-Saharan Africa by country name: Africa OR Algeria OR Angola OR Benin OR Botswana OR Burkina Faso OR Burundi OR Cameroon OR Canary Islands OR Cape Verde OR Central African Republic OR Chad OR Comoros OR Democratic Republic of Congo OR Djibouti OR Egypt OR Equatorial Guinea OR Eritrea OR Ethiopia OR Gabon OR Gambia OR Ghana OR Guinea OR Guinea OR Guinea Bissau OR Ivory Coast OR Kenya OR Lesotho OR Liberia OR Libya OR Libi OR Libia OR Madagascar OR Malawi OR Mali OR Mauritania OR Mauritius OR Morocco OR Mozambique OR Mocambique OR Namibia OR Niger OR Nigeria OR Principe OR Reunion OR Rwanda OR Sao Tome OR Senegal OR Seychelles OR Sierra Leone OR Somalia OR South Africa OR St Helena OR Sudan OR Swaziland OR Tanzania OR Togo OR Tunisia OR Uganda OR Western Sahara OR Zaire

The search terms for the outcome of interest were ‘oropharyngeal’, ‘candidiasis’, ‘OPC’, ‘Oral thrush’, ‘Candida’, ‘Non Albicans Candida’, ‘NAC’, ‘non *Candida albicans*’, Candida’, ‘*C. albicans*’, ‘*C. parapsilosis*’, ‘*C. glabrata*’, ‘*C. tropicalis*’, ‘*C. dubliniensis*’, *C.krusei*’, ‘*C. norvegensis*’, ‘ *C. guilliermondii*’, *C. albicans*’, ‘*C. glabrata*’, ‘*C. tropicalis*’, ‘*C. krusei*’, ‘*C. dubliniensis*’, ‘*C. parapsilosis*’, ‘*C. guilliermondii*’, ‘*C. famata*’, ‘*C. kefyr*’, ‘*C. norvegensis*’, ‘*C. sake*’, ‘*C. lusitaniae*’, ‘*C. pintolopesii*’, ‘*C. pseudotropicalis*’, ‘*C.globosa*’, ‘*C. dattila*’, ‘*C. inconspicua*’, ‘*C. hellenica*’, ‘*C. holmii*’, ‘*C. pulcherrima*’, ‘ *C. valida*’, ‘*C. africana*’, ‘*C. fabianii*’, ‘*C. cacaoi*’, ‘*C. zeylanoides*’.

The search terms for study design were ‘cross sectional’, ‘observational’, ‘descriptive’, ‘prevalence’, ‘transverse’, ‘cohort’, and ‘case control’.

Specific terms for the intervention and comparator were not applied since this review was neither an intervention nor a comparison study. This search was restricted to the period from 1^st^ January 2000 to 8^th^ October 2022. In addition, the reference lists of all included studies were searched, and gray literature was searched on Google Scholar for more articles.

### Review question and eligibility criteria

The review question was “What is the prevalence of oropharyngeal candidiasis and the distribution of *Candida* species among people living with HIV in Africa?” This question was described further (Table 1) using the PICOST framework, which guided the eligibility of the included studies. Studies were included if they were published in the English language between 1^st^ January 2000 and 8^th^ October 2022 and if they reported outcomes of interest, that is, the prevalence of OPC and distribution of *Candida* species causing OPC among PLHIV in Africa. This review included only observational studies with either cross-sectional, case‒control or cohort study designs reporting OPC and *Candida* species among PLHIV living in Africa. We included studies that diagnosed OPC infection based on both the presence of oral lesions and the mycological identification of *Candida* species isolated from the oral cavity of PLHIV. We excluded studies that reported the prevalence of OPC without information on the causative Candida species. We excluded animal model reports and observational studies whose full text could not be retrieved even after request from the corresponding authors and comprehensive library search.

### Study outcomes

The primary study outcome was the prevalence of OPC in PLHIV. The secondary outcome was the distribution of *Candida* species (Table 1).

### Study selection process

#### Data management

**Figure 1:**
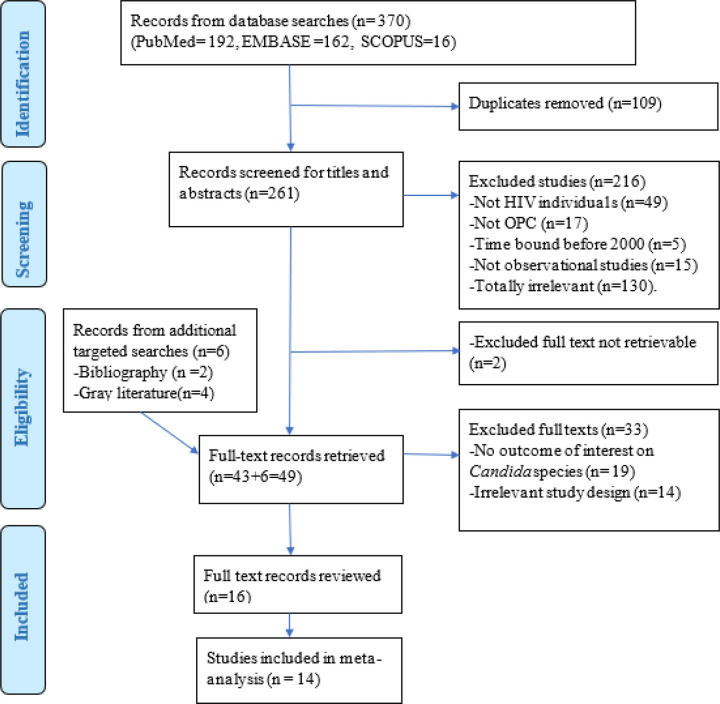
PRISMA flowchart showing the literature search and selection process

Using Mendeley Desktop referencing software version 1.19.8 (Mendeley Ltd., London, UK), we imported all identified titles, excluded duplicates, and screened and grouped these into relevant eligibility categories as described in our Preferred Reporting Items for Systematic Reviews and Meta-analyses (PRISMA) flow chart (Figure 1).

#### Minimizing bias in study identification and selection

Two reviewers (BM and AAK) carefully conducted the literature search. Two independent reviewers (HI and GM) examined relevant studies and screened their titles and abstracts for eligibility. After the initial screening, the full texts of the eligible studies were retrieved and examined for eligibility by RN and AM. Disagreements were resolved by discussion with two reviewers (BA and OJS) to reach consensus.

#### Data extraction

Data extraction was performed using a spreadsheet developed from Microsoft Excel version 16 (Microsoft Corporation, Richmond, Seattle, Washington, USA). The extracted data included the first author, year of publication, country where the study was conducted, study design, sample size, gender, mean age of the study population, *Candida* identification method, and prevalence of OPC and *Candida* species. The data were extracted in duplicate by BM and RN, and any disagreements were resolved by a third party (AM).

#### Qualitative assessment

Two reviewers (GM and BM) independently assessed the risk of bias of the included studies, and any discrepancies between the two reviewers were resolved by reaching a consensus through discussion. Eligible studies were assessed for risk of bias using the Joanna Briggs Institute quality assessment tool for prevalence studies (16). This tool consists of 9 parameters: (1) an appropriate sampling frame to address the target population, (2) a proper sampling method, (3) an adequate sample size, (4) a description of the study subject and setting, (5) sufficient data analysis, (6) the use of valid methods for the identified conditions, (7) valid measurements for all participants, (8) the use of appropriate statistical analysis, and (9) an adequate response rate. Each parameter was scored either 1 for failure to answer the question or 0 for ability to answer the question. The risk of bias was classified as low, moderate, or high, with total scores ranging from 0 to 2, 3 to 4 or 5 to 9, respectively (Table 4).

### Synthesis

The extracted data were imported into STATA 17.0 statistical software (STATA, College Station, Texas) for analysis. Descriptive statistics and narrative synthesis were used to summarize the data and present the results from the included eligible studies, respectively. A random effects cumulative meta-analysis was performed using STATA 17.0 to map the prevalence of OPC among PLHIV for each study and estimate the summary estimate (meta-analysis) in Africa. These results are displayed in a forest plot. We visually explored heterogeneity by inspecting the forest plot and statistically quantifying it using the I^2^ statistic. Because we found a high level of heterogeneity, we conducted a meta-regression testing the variables of year of publication and sample size before conducting a sensitivity analysis by a leave-one-out sensitivity analysis. Additionally, publication bias was evaluated using funnel plots and Egger’s test at a significance level of < 0.05. This sensitivity analysis explored the significant differences between the distribution of *Candida* species and the prevalence of OPC among people living with HIV in Africa. Any value with *P* < 0.05 was considered statistically significant at the 95% confidence interval (CI).

### Sensitivity analysis

To identify the source of heterogeneity, a leave-one-out sensitivity analysis was employed. Sensitivity analysis was performed using the random effects model.

### Meta-regression

Meta-regression analysis was performed to explore the associations between prevalence of OPC and the year of publication and study sample size.

## RESULTS

### Search results

The PRISMA flow chart summarizes the identified, screened, excluded, and included studies with reasons for exclusion. The database search yielded 370 titles from the PubMed (n=192), EMBASE (n=162) and SCOPUS (n=16) databases. After removing duplicates (n=109), 261 titles or abstracts were screened, and 216 studies were excluded, mainly due to irrelevance (n=130), not including PLHIV (n=49) and OPC (n=17), non-observational study design (n=15) or a time limit (n=5). During full-text retrieval, 2 articles were not retrievable and were excluded. However, 49 full-text records were retrieved, including 6 from gray literature (n=4) and reference lists of included studies (n=2). The excluded full-text studies (n=33) lacked information on the distribution of *Candida* species (n= 19) or had in appropriate study designs (n=14). Sixteen studies were reviewed, and 14 meta-analyses were performed using a random effects model.

### Summary of included studies

Sixteen (16) observational studies reporting *Candida* species causing OPC in 9 countries were fully reviewed and included in the qualitative synthesis. These studies were conducted in Nigeria (n=3) (17–19), Cameroon (n=3) (20–22), South Africa (n=3) (21,23,24), Ethiopia (n=2) (25,26), Uganda (n=1) (7), Tanzania (n=1) (27), Ghana (n=1) (28), Chad (n=1) (29) and the Ivory coast (n=1) (30) (Table 2). Whereas 14 studies reported on both the distribution of *Candida* species and the prevalence of OPC, 2 studies did not report on the exact prevalence of OPC but reported on the distribution of *Candida* species (24,27). All 16 studies had a total sample size of 4,485 participants. The largest study had a sample size of 605 participants, while the smallest study had 197 participants (Table 2). A total of 2,688 *Candida* isolates were reported in 9 countries, with the majority from South Africa (n=473) and Cameroon (n=409), while Ghana (n=201) and the Ivory coast (n=227) had the lowest frequencies (Figure 3).

### Findings on the outcomes of interest

#### Prevalence of oropharyngeal candidiasis (OPC) among people living with HIV

**Figure 2:**
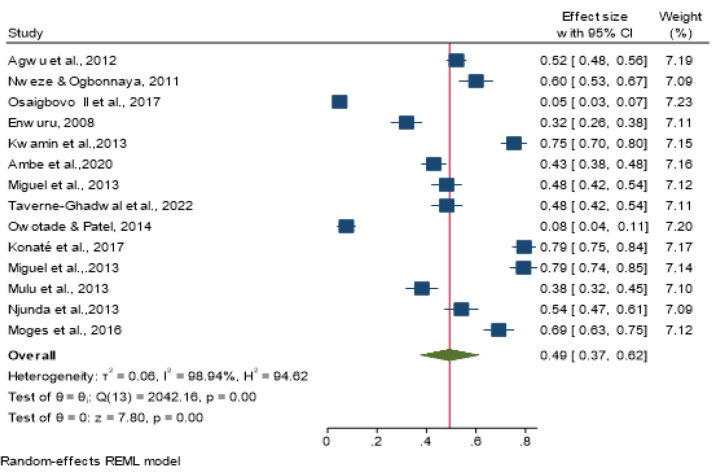
The prevalence of oropharyngeal candidiasis, 95% CI, and heterogeneity (I^2^) across different studies according to the random effects model (REML)

We included 14 of the 16 review studies in the meta-analysis for the primary outcome. All 14 studies were conducted in 9 African countries and clearly demonstrated a sample size of 3863 participants. The prevalence of OPC ranged from 4.9% in Nigeria to 79.4% on the Ivory coast (18,30) (Table 2). The pooled (average) prevalence was 49.0% (1887/3863), with a 95% CI of 37 to 62%. There was high heterogeneity across the studies (*I*^2^, 98.94%; *I*^2^>75%), with a P value< 0.001 according to the random effects model (Figure 2). Two studies were within the funnel plot (Figure 5). Three (3) studies conducted in Nigeria and Cameroon reported prevalence rates of 60%, 4.9%, and 31.9% (17–19) and 43%, 48%, and 54.1% (20–22), respectively. In addition, 2 studies conducted in South Africa and Ethiopia reported a prevalence of 7.6% and 79.4% [27], [30] and 54.1% and 69.2% (25,26), respectively, while a prevalence of 52%, 75.3%, 34% and 79.4% was reported in Uganda, Ghana, Chad and the Ivory coast, respectively [14], [25], [29], [32] (Table 2).

#### Distribution of Candida species among people living with HIV with OPC

**Figure 3:**
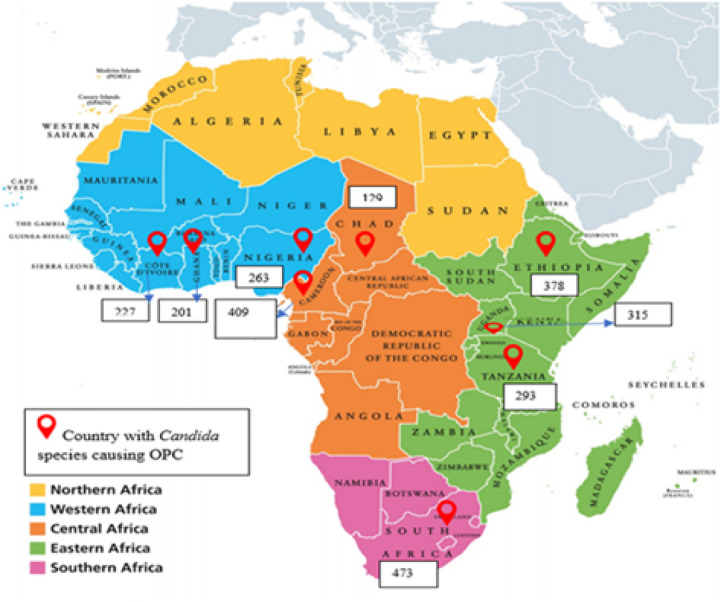
Map of Africa showing the frequency of Candida isolates from individuals with HIV with OPC in 9 countries.

In the 16 reviewed studies conducted in 9 countries, a total of 2,688 *Candida* isolates were reported to cause OPC among people living with HIV in 9 countries in Africa. The most burdened countries were South Africa (n=473) and Cameroon (n=409), while Ghana (n=201) and the Ivory coast (n=227) had the lowest frequencies (Figure 3). Among the 2688 *Candida* isolates, 76.6% (n=2060) were *Candida albicans*, 21.7% (n=582) were NAC and 1.7% (n=46) were unidentified *Candida* species. *The prevalence of C. albicans ranged from 55.1% (n=145) in Nigeria to 95.2% (n=216) in Ivory coast, while the prevalence of NAC ranged from 5.4% (n=17) in Uganda to 43.7% (n=115) in Nigeria. In addition to C. albicans and NAC, the prevalence of unidentified Candida species ranged from 0.4% in Cameroon to 7.6% in Uganda (Table 3)*. Among the 582 NAC species, *C. glabrata* was the most prevalent species (29.6%; 172/582), followed by *C. tropicalis* (27.7%; 161/582), *C. krusei* (17.0%; 99/582), *C. parapsilosis* (8.1%; 47/582) and *C. dubliniensis* (5.2%; 30/582). Some species were rare and were found only in single countries; for example, *C.dattila, C.hellenica, C.holmii, C.pulcherrima and C.valida* were found only in Ghana; *C.fabiani and C.cacaoi* were found only in Chad; and *C.zeylanoides* were found only in Cameroon (Table 3).

Among the 46 unidentified *Candida* species, Uganda had the greatest number (52.2%; 24/46).

#### Trends in the Cumulative Prevalence of C. albicans, NAC and OPC

**Figure 4:**
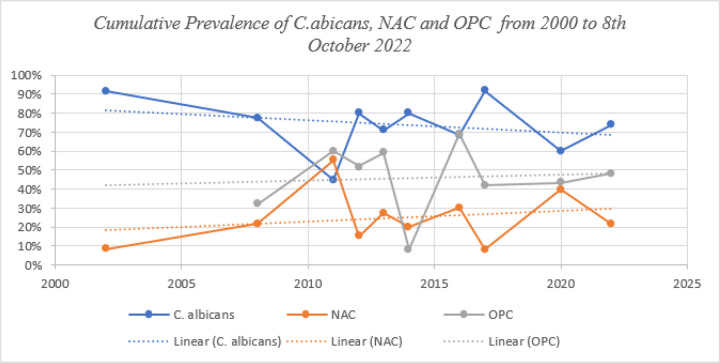
Cumulative prevalence of Candida species and OPC from 2000 to 8^th^ October 2022 Legend: NAC=Non *albican candida*, OPC= oropharyngeal candidiasis.

**Figure 5:**
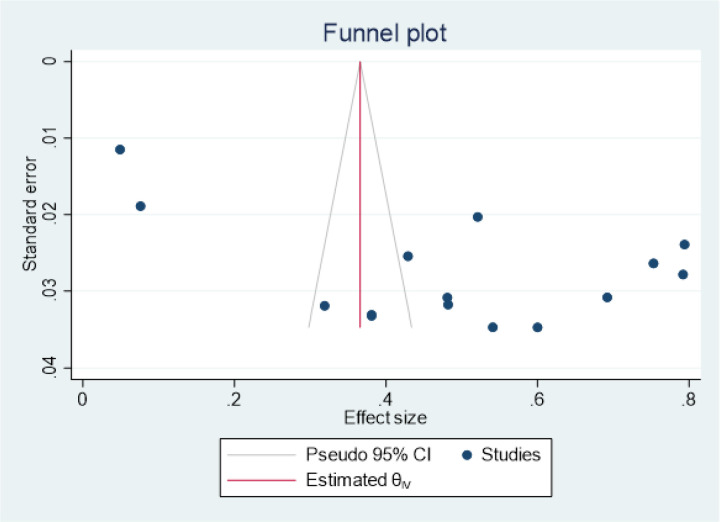
A funnel plot showing publication bias of studies included for the prevalence of OPC among PLHIV in Africa

Generally, the prevalence of NAC and OPC has *increased over the past two decades, while that of C. albicans has decreased slowly (Figure 5). The cumulative prevalence of C. albicans ranged from 45% in 2011 to 92% in 2017, from 8% in 2002 to 55% in 2011 for NAC, and from 32% in 2014 to 69% in 2016 for OPC* (Figure 4).

### Risk of bias in the included studies

According to the risk of bias assessment criteria used, seven studies (7/14: 50.0%) had a low risk of bias, five (5/14: 35.7%) had a moderate risk of bias, and two (2/14: 14.3%) had a high risk of bias, as shown in Table 4. Most of the included studies had a potential risk of bias due to the methods used in the diagnosis of OPC infection across study participants (10/14: 71%).

### Heterogeneity

There was high heterogeneity across the studies (*I*^2^, 98.94%; *I*^2^>75%), with a P value< 0.001 according to the random effects model (Figure 2). Two studies were within the funnel plot (Figure 5).

### Risk of publication bias

Publication bias was assessed based on asymmetry of the funnel plot and statistically using Egger’s test at a significance level of < 0.05. According to the funnel plot, the majority of the studies were outside (n=12), and there was a slight visual publication bias (Figure 5). Egger’s test (Ho: beta1 = 16.37, P=0.06) was also performed. However, we failed to reject the null hypothesis (Intercept B1=0); hence, there was no publication bias.

### Sensitivity analysis

To identify the source of heterogeneity, a leave-one-out sensitivity analysis was employed. Sensitivity analysis was performed using the random effects model, which showed that there was no single study that influenced the overall prevalence of OPC, as shown

### Meta-regression

**Figure 6:**
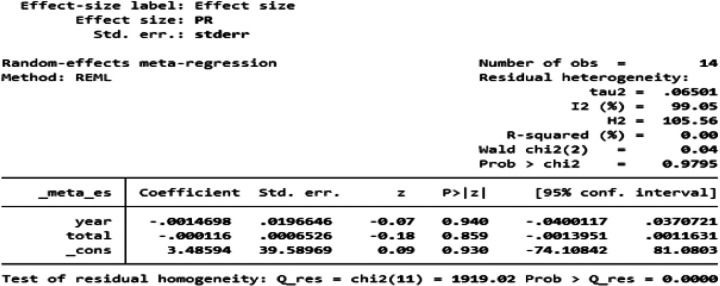
A meta-regression analysis showing the effect of the year of publication and sample size on the prevalence of OPC

According to our meta-regression analysis, the year of publication and sample size were not significant sources of heterogeneity for the prevalence of OPC. This study revealed no significant associations between of OPC and the year of publication (p value = 0.940) or study sample size (p value = 0.859) (Figure 6).

## DISCUSSION

### Principal findings

This systematic review and meta-analysis aimed to determine the prevalence of OPC and the distribution of *Candida* species among PLHIV in Africa. The main findings indicate a pooled prevalence of 49.0%, and the prevalence ranged from 4.9–79.4% in different African countries. Among the total *Candida* isolates (n = 2,688) from PLHIV in Africa, approximately 76.6% (n = 2,060) were *Candida albicans*, and 21.7% (n = 582) were non-*Candida albicans*. Among the non-*Candida albicans* species, *C. glabrata* was the most common isolate (29.6%), followed by *C. tropicalis* (27.7%), *C. krusei* (17.0%), *C. parapsilosis* (8.1%) and *C. dubliniensis* (5.2%). South Africa had the highest frequency of *Candida* isolates (n = 473), while Ghana had the lowest (n = 201). Our results reinforce reports in several studies regarding the increase in OPC and NAC species. This contributes to the increasing evidence that there is increased OPC among PLHIV and a shift in OPC etiology towards NAC species, hence justifying the need for laboratory diagnosis of OPC and speciation of *Candida* species to improve its diagnosis and management (12–14, 31, 32).

### Findings in relation to other reviews

The findings of our primary outcome reinforce other reviews reporting a higher of OPC among PLHIV in Africa. For instance, a study by Tappuni and colleagues reported that the prevalence of OPC was 52% in Africa, 39% in Asia and 30% in America (33). Furthermore, our pooled prevalence of 49.0% was consistent with studies conducted in Indonesia and Iran that reported that the prevalence of OPC among PLHIV was 46.2% and 59.3%, respectively (34, 35). However, our prevalence was higher than the 23.6% reported in South Asia (34), 26.1% in China (36) and lower than the 80.6% reported in India (37). The difference in prevalence across different countries and continents may be due to variations in oral hygiene, diagnostic approaches, CD4 levels, availability of HAART, treatment of candidiasis and geographic location (38). Owing to the high burden of HIV/AIDS, PLHIV in Africa have an increased risk of OPC caused by a wide variety of *Candida* pathogens. A shift in the etiology associated with OPC towards antifungal-resistant NAC in Africa could explain the increase in cumulative prevalence, which increased from 32% in 2014 to 69% in 2019 and then decreased to 48% in 2022 (26, 39, 40). Immunosuppression characterized by reduced CD4 + T lymphocytes and HAART failure leading to a high viral load among PLHIV is a probable consequence of increased antifungal NAC (26, 28, 41–43).

Generally, *C. albicans* (76.6%) was the most common species reported to cause OPC among PLHIV compared to NAC (21.7%). The higher frequency of *C. albicans* was in agreement with other studies that found *C. albicans* to be the dominant *Candida* species causing OPC in China (71%), Indonesia (50%), India (50%) and Iran (58%) (36, 44–46). Owing to its stronger ability to adhere to buccal epithelial cells and form complex biofilms, *C. albicans* is isolated more frequently than other NAC species that cause OPC (6, 47, 48). Although the high percentage of *C. albicans* associated with OPC may be a reflection of its virulence, it is possible that its high prevalence could be due to misdiagnosis caused by the use of conventional phenotypic and biochemical methods, which have reduced the sensitivity of detecting NAC (49).

In addition, *C. albicans* was the most isolated *Candida* species, and other NAC species (n = 582, 21.7%) were identified as causes of OPC among PLHIV in Africa. The predominant NAC species were *C. glabrata* (29.6%), *C. tropicalis* (27.7%), *C. krusei* (17.0%), *C. parapsilosis* (8.1%) and *C. dubliniensis* (5.2%). Our results support observations that have been reported in several other studies regarding the epidemiological shift of OPC etiology toward NAC species, and this has accounted for their emergence as a significant *Candida* pathogen (26, 39, 40). For instance, the distribution of NAC species in our study agreed with studies conducted in Indonesia, Iran and India that identified *C. glabrata* (15–19%), *C. krusei* (4.6–15%) and *C. tropicalis* (4.6–10%) as the most prevalent NAC species (44–46). This shift may be due to the use of antimicrobial agents, such as antifungals, antiretroviral and antibiotics (2). Exposure to these agents may exert positive selection pressure on NAC species, which are considered intrinsically resistant to antifungal agents, thus increasing their prevalence (11, 50). In addition, recent studies have demonstrated that *C. albicans* and *C. glabrata* have a synergistic relationship in which *C. albicans* facilitates the initial development of OPC infection by *C. glabrata* (6, 51).

Approximately 1.7% (n = 46) of the isolates could not be identified up to the species level, and Uganda reported the highest percentage of unidentified *Candida* species (52.2%; 24/46). This is not surprising since accurate identification of *Candida* species has been reported as a challenge in Uganda, which requires better laboratory approaches for definitive diagnosis of candidiasis (7, 31, 32, 52, 53).

Our findings support studies that have reported an increase in OPC and an etiological shift towards NAC species. Hence, accurate laboratory diagnosis of OPC and speciation of *Candida* species are needed to improve its diagnosis and management (12–14, 31, 32).

### Implications of this review for health professionals, future research and policy

Owing to increased OPC coupled with both *C. albicans* and NAC among PLHIV in Africa, clinicians and laboratory professionals or microbiologists should use accurate molecular diagnostic approaches to differentiate *Candida* species and their antifungal susceptibility profiles. This would be a long way from decreasing the morbidity associated with OPC.

Although an increase in emerging NAC species has been noted in this review, we did not look at the virulence attributes and antifungal resistance patterns of *Candida* species. Understanding virulence factors is vital for understanding OPC pathogenesis and consequently helps improve the diagnosis and therapeutic treatment of OPC among PLHIV. This is an area that can be strengthened in future studies.

Policy makers and actors should invest in mycology laboratories and support research in this area.

### Limitations of this review and meta-analysis study

The strengths of this review and meta-analysis study were the use of a rigorous search of the PubMed, Scopus, and EMBASE databases following the PRISMA statement. However, this meta-analysis has the following limitations. The studies included in this review had a wider range of OPC prevalence (4.9–79.4%), meaning that some studies had a low prevalence, while others had a high prevalence. We found only 14 articles that were used in the meta-analysis, and we excluded studies that did not include speciation for *Candida*. Additionally, papers that were not written in English were excluded. This may affect the estimated pooled prevalence of OPC among PLHIV in Africa.

## Conclusion

Almost half of PLHIV in Africa have OPC. *C. albicans* remains the most common etiology of OPC in this cohort of individuals. However, there is a significant shift in the etiology towards NAC species.

## Data Availability

The datasets analyzed during the current study are available from the corresponding author upon reasonable request.
